# Supplementary material for: Splice-Junction-Based Mapping of Alternative Isoforms in the Human Proteome
Source: Cell Rep. Author manuscript; Available in PMC 2020 Jan 15. (PMC6961840; doi:10.1016/j.celrep.2019.11.026)
Supplement: 3 [file NIHMS1546469-supplement-3.zip › DF2/PXD006675/CardiacFibroblast-86-Q5VZF2-ALTSAQLQQHAAFIPTDNSEIISR.pdf]

A

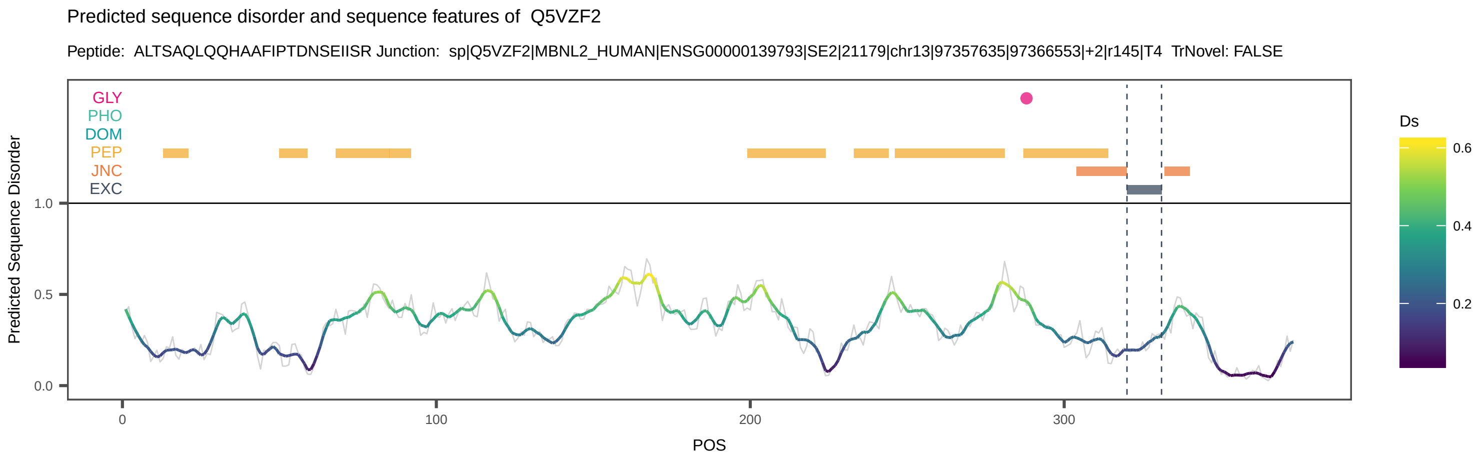

Predicted sequence disorder and sequence features of Q5VZF2
Peptide: ALTSAQLQQHAAFIPTDNSEIISR Junction: sp|Q5VZF2|MBNL2_HUMAN|ENSG00000139793|SE2|21179|chr13|97357635|97366553|+2|r145|T4 TrNovel: FALSE
GLY
PHO
DOM
PEP
JNC
EXC
Predicted Sequence Disorder
1.0
0.5
0.0
0
100
200
300
POS
Ds
0.6
0.4
0.2


B

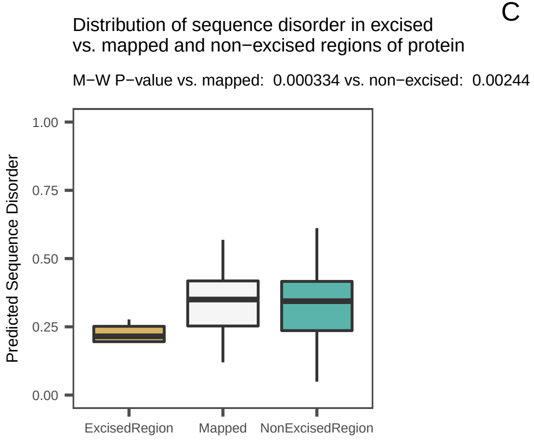

Distribution of sequence disorder in excised
vs. mapped and non−excised regions of protein
M−W P−value vs. mapped: 0.000334 vs. non−excised: 0.00244
Predicted Sequence Disorder
1.00
0.75
0.50
0.25
0.00
ExcisedRegion
Mapped
NonExcisedRegion


C
